# The attitude of patients with progressive ataxias towards clinical trials

**DOI:** 10.1186/s13023-021-02091-x

**Published:** 2022-01-04

**Authors:** Gilbert Thomas-Black, Andrada Dumitrascu, Hector Garcia-Moreno, Julie Vallortigara, Julie Greenfield, Barry Hunt, Susan Walther, Mackenzie Wells, David R. Lynch, Hugh Montgomery, Paola Giunti

**Affiliations:** 1 Department of Clinical and Movement Neurosciences, The Ataxia Centre, UCL Queen Square Institute of Neurology, University College London, London, UK; 2 National Hospital for Neurology and Neurosurgery, University College London Hospitals Foundation NHS Trust, London, UK; 3 Ataxia UK, 12 Broadbent Close, N6 5JW, London, UK; 4 Friedreich‘S Ataxia Research Alliance, 533 W Uwchlan Ave, Downingtown, PA 19335 USA; 5 Departments of Pediatrics and Neurology, Children’s Hospital of Philadelphia, Philadelphia, PA 19104 USA; 6 Institute of Sport Exercise and Health, University College London, London, UK

**Keywords:** Clinical trials, Patient attitude, Ataxias, Trial design, Trial participation, Trial investigations, Time commitment, Drug administration

## Abstract

**Background:**

The development of new therapies may rely on the conduct of human experimentation as well as later clinical trials of therapeutic interventions. Ethical considerations seek to protect the patient from risk but few have sought to ascertain the attitude to such risk of patients with progressive debilitating or terminal conditions, for which no mitigating or curative therapies exist. Such understanding is also important if recruitment is to be maximized. We therefore sought to define the motivations for and barriers to trial participation amongst patients with progressive ataxias, as well as their condition-specific trial preferences.

**Methods:**

We conducted an online survey consisting of 29 questions covering four key domains (demographics, personal motivation, drug therapy and study design) relating to the design of clinical trials. Two major ataxia charities, Ataxia UK and the Friedreich’s Ataxia Research Alliance (FARA) sent the survey to their members. Responses were analysed by disease and by ambulatory status.

**Results:**

Of 342 respondents, 204 reported a diagnosis of Friedreich’s ataxia (FRDA), 55 inherited cerebellar ataxia (CA) and 70 idiopathic CA. The most important symptoms to be addressed by a trial were considered to be balance problems and ambulation, although these were superseded by speech problems in wheelchair users. Common motivations for participation were potential benefits to self and others. Reasons for non-participation included concerns about side effects, and the burden and cost of travel. Financial reimbursement for expenses was reported to be likely to increase trial engagement, Phase two trials were the most popular to participate in, and the use of a placebo arm was seen as a disincentive. Across all disease subgroups, drug repurposing trials proved popular and just under 70% of participants would be prepared to undergo intrathecal drug administration.

**Conclusions:**

Knowledge of motivations for and barriers to trial participation as well as the acceptability of investigations, time commitments and routes of drug administration should inform better, more patient focused trial design. This in turn may improve recruitment and retention of participants to future trials.

**Supplementary Information:**

The online version contains supplementary material available at 10.1186/s13023-021-02091-x.

## Background

Ataxia may result from cerebellar or sensory impairment, and may be acquired (e.g. due to alcohol excess) or inherited. Additional neurological features (e.g. extrapyramidal signs or spasticity) or pathology outside the neuraxis (e.g. the cardiomyopathy seen in Friedreich’s ataxia (FRDA)) may also be present. Mobility and communication skills are profoundly reduced, impacting quality of life, with many ataxias also leading to premature death [1, 2].

The inherited ataxias are a large group of degenerative and metabolic disorders of enormous clinical and genetic heterogeneity. Epidemiological studies report an estimated overall prevalence rate of 26/100,000 in children, of 2.7/100,000 and 3.3/100,000 for dominant hereditary cerebellar ataxia, recessive hereditary cerebellar ataxia respectively [3]. Diagnosis can be challenging and there are a number of people who are classified as having idiopathic cerebellar ataxia. Currently, there are few disease-modifying agents available, and most are of limited impact [4].

The design of trials in rare diseases is often challenging: patient numbers are low, phenotypic expression often variable and disease time-course lengthy and trajectory poorly mapped, all factors which hinder identification of therapeutic subgroups and powering against specific endpoints. The ataxia community has made great effort to address these issues. In FRDA, the commonest inherited ataxia, the Friedreich Ataxia Clinical Outcome Measure Study (FACOMS) [5] and European Friedreich’s Ataxia Consortium for Translational Studies (EFACTS) [6] have provided data on the natural history of FRDA for up to 12 years. The EUROSCA Natural History Study provides similar data for the commonest autosomal dominant spinocerebellar ataxias [7]. These studies have validated measures of neurological change, quality of life and disability and also act as international disease registries enabling facilitation of multi-centre trials which have improved enrolment. The recruitment of specific subgroups of patients, e.g. early- versus late-stage, may be important for a trial depending on the trial's chosen outcome measure. For example, in FRDA progressive difficulties with ambulation are seen early in the course of the disease [8], therefore a trial with measure of ambulatory function as its primary outcome measure should aim to recruit participants from this subgroup. Thus, it is important to understand what drives patients at different stages of the disease to participate in trials.

European data relating to change in symptom scores (e.g. the Scale for Assessment and Rating of Ataxia (SARA) score [9] or Friedreich’s Ataxia Rating Scale (FARS) [10]) suggest that to detect a reduction of 50% in SARA progression at 80% power, 548 patients would be required for a 1-year trial and 184 in a 2-year one [11]. American studies support such estimates of powering [5]. There is thus a need to increase the available pool of potential participants, to develop more sensitive measures that would facilitate short-term trials, and to identify robust and sensitive biomarkers of disease activity and progression, such that study power can be increased, sample size reduced, and trial duration shortened.

For now, these barriers limit the ability to test novel therapies. This is especially true where ‘first into man’ studies carry risk. For patients, however, the absence of intervention can lead to irreversible decline in function and, ultimately death, and it may be that their appetite for risk is greater than that of ethics committees reviewing trial protocols. In general, patient-centred approaches to study design and execution are likely to yield more successful trials [12]. Engagement can help identify patients’ motivations for and obstacles to participation, and also lead to improved trial design and acceptability.

Here, we present the results of an electronic survey designed to explore the ataxic patient’s perspective on these complex issues and discuss their implications for future trials.

## Results

### Cohort demographics

**Fig. 1:**
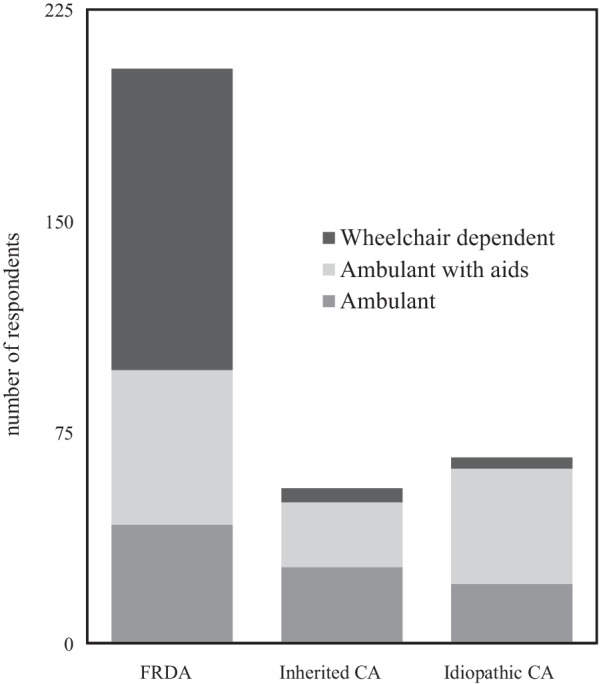
Survey respondents by condition and ambulatory status

In total, there were 342 respondents to the survey (demographic details supplied in Table 1) of whom 204 reported a diagnosis of FRDA, 55 inherited CA and 70 idiopathic CA (Fig. 1). Respondents were predominantly (77.2%) patients, with parents (15.8%), carers (5%) and partners (2%) also completing the survey on the patients’ behalves. The majority (87.7%) of FRDA respondents were from the USA, with the UK providing the majority of inherited CA (98.1%) and idiopathic CA (91.4%) cases.

**Table 1:** Survey respondent demographics

*Respondent type (total number of respondents* = *342)*	n (%)
Patient	264 (77.2%)
Parent	54 (15.8%)
Carer	17 (5%)
Partner	7 (2%)
*Age distribution (n* = *338)*	
Under 15	3 (0.9%)
15–25	50 (14.8%)
26–35	57 (16.9%)
36–45	53 (15.7%)
46–55	62 (18.3%)
56–65	58 (17.2%)
> 65	55 (16.3%)
Female sex	62%
UK-based	45%
*Condition*	
FRDA	204 (59.6%)
Inherited CA	55 (16%)
Idiopathic CA	70 (20.4%)
Episodic ataxia	12 (3.5%)

*CA*: cerebellar ataxia, *FRDA*: Friedreich’s ataxia

### Symptoms to be targeted in future trials

Respondents were asked to identify the top three symptoms (of 16 options) that they would most like to see addressed by a clinical trial (Additional file 1: Appendix 1). The top five most frequently selected by the FRDA cohort were problems with walking (51.3%), balance problems/unsteadiness (47.3%), fatigue (34%), slurred speech (31.3%) and dexterity (29.3%). Analysis by ambulatory status subgroup demonstrated that patients who were wheelchair dependent selected slurred speech (41.5%) as the symptom they would most like addressed by a trial, while both subgroups of ambulatory patients selected balance problems (walking independently, 75% and walking with aids, 67.5%). The top five most frequently selected by inherited CA and idiopathic CA respondents had similar priorities with balance problems/unsteadiness (83.3% and 82.5% respectively), walking (47.2% and 35%), slurred speech (38.9% and 30%), fatigue (22% and 32.5%), and dexterity (13.9% and 15%) (Additional file 2: Table S1).

### Motivations and barriers to trial participation

Respondents were asked what were their top six motivating factors for, and barriers to, joining a clinical trial (Additional files 3 and 4: Tables S2 and S3) from a list provided in the survey (Additional file 1: Appendix 1). Key motivating factors for the FRDA cohort were potential benefit to self (92%), potential benefit to others (80.2%), financial reimbursement of expenses i.e. covering costs of travel and accommodation if applicable (63%), recommendation of trial by physician (51.2%) and availability of a physician should there be any issues (49.4%). For inherited CA, the top factors were potential benefit to self (88.1%), potential benefit to others (85.7%), availability of a physician should there be any issues (57.1%), recommendation of the trial by a physician and a feeling of increased care/obtaining extra health check-ups (both 35.7%). Major deterrents for FRDA patients were costs associated with travel (67.9%), burden of travel (58%), fear of side effects (50.6%), stopping current regular medication (43.2%) and having to miss work/school (38.9%). For inherited CA the top five deterrents were fear of side effects (65.9%), costs associated with travel (48.8%), burden of travel (46.3%), worry that trial medication is not effective (31.7%) and stopping current regular medication (22%). For idiopathic CA, the main deterrents were fear of side effects (65.3%), burden of travel (61.2%), costs associated with travel (42.9%), not being able to take future medication due to potential interactions with study drug (28.6%) and stopping current regular medication (26.5%).

### Interest in future trial participation

Across the cohort, FRDA respondents were most interested in participating in future trials (73.9%), ahead of those with inherited ataxia (65.2%) and idiopathic ataxia (59.2%). We explored factors influencing interest in trial participation, hypothesising that people who had previously taken part in a trial would be more likely to participate in a future one. Survey responses (n = 255) were dichotomised into very and extremely interested in joining a future trial (n = 207) and all other responses (n = 91) and a binary logistic regression model fitted. The only covariate that proved statistically significant was having previously taken part in more than one trial (OR = 2.761, 95% CI 1.208, 6.311, *p* = 0.016), thus confirming our hypothesis (Additional file 3: Table S2 and Additional file 4: Table S3).

### Trial design factors influencing trial participation

**Fig. 2:**
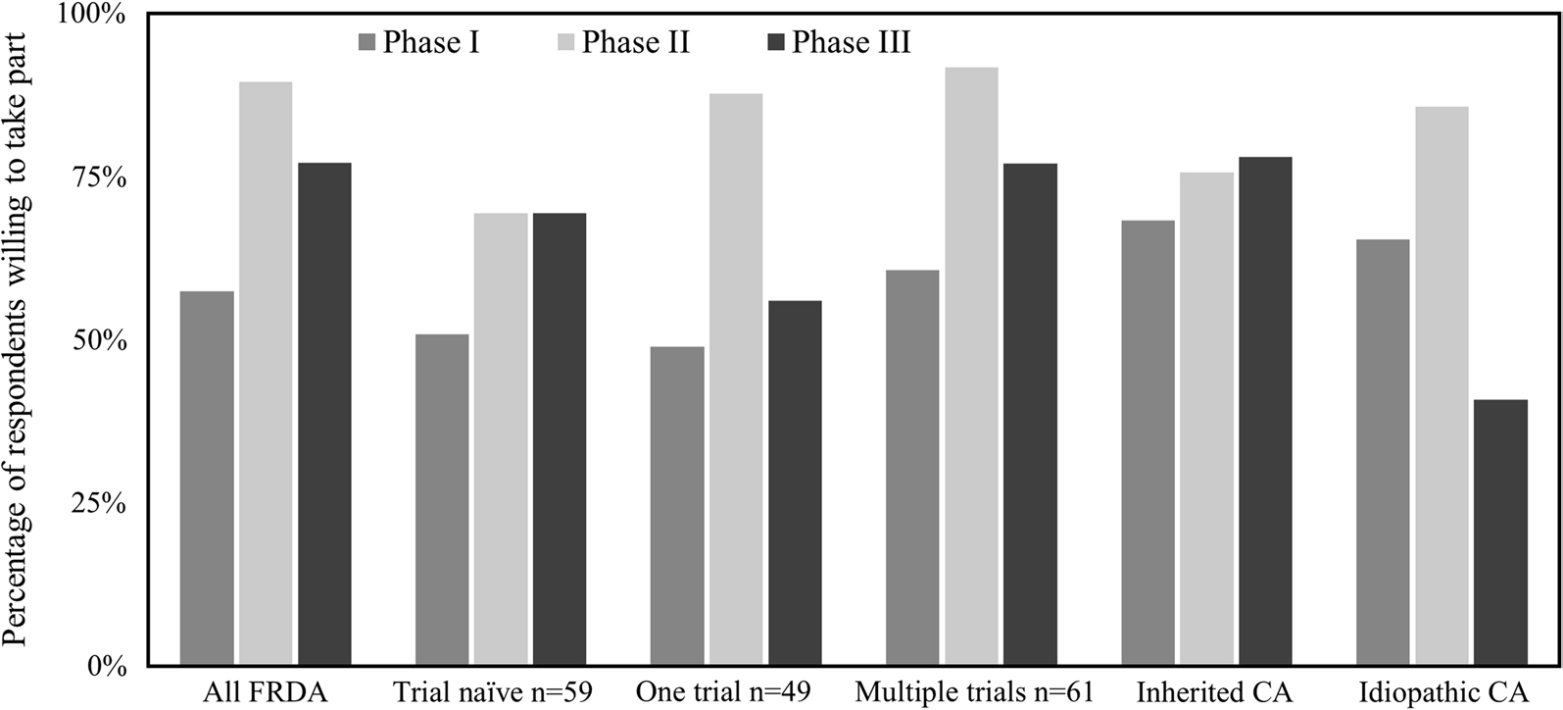
Percentage of respondents willing to take part in different trial phases. Columns represent condition and trial status (if FRDA respondent)

The most popular phase of trial to participate in for FRDA and idiopathic CA respondents was phase 2 (89.5% and 85.7% respectively), and phase 3 for inherited CA respondents (78%) (see Additional file 1: Appendix 1 for phase definitions). Of FRDA respondents, 57.4% would take part in a phase 1 study, compared to 68.3% of inherited CA respondents and 65.3% of idiopathic CA respondents (Fig. 2, Table 2). Use of placebo proved to be a disincentive for participants; the percentage of respondents who would be very or extremely likely to participate in a clinical trial if it was placebo controlled was 59.5% for FRDA, compared to 73.9% to who would be very or extremely interested in participating in a future trial in general. Similar trends were seen in inherited CA, 48.9% versus 65.2% and for idiopathic CA, 20.8% versus 59.2%. We then looked to see if the aversion to placebo was mitigated by the option of an open label extension phase. Here, 68.9% of FRDA respondents stated they would be very or extremely more willing to participate, compared with 47.8% of inherited CA and 37.7% of idiopathic CA respondents (Table 2).

**Table 2:** Respondents views on trial design

	FRDA all (%)	FRDA trial naive (n = 59) (%)	FRDA one trial (n = 49) (%)	FRDA multiple trials (n = 61) (%)	Inherited CA (%)	Idiopathic CA (%)
Respondents who are trial experienced	63.9				12.7	4.5
Respondents very or extremely interested in participating in a future clinical trial	73.9	70.1	67.3	86.7	65.2	59.2
Respondents very or extremely interested in participating in a future clinical trial (drug repurposing)	75.2	74.1	73.4	81.9	53.1	50.9
Respondents willing to participate in phase of trial						
Phase 1	57.4	50.8	48.9	60.6	68.3	65.3
Phase 2	89.5	69.4	87.7	91.8	75.6	85.7
Phase 3	77.1	69.4	55.9	77	78	40.8
Respondents very or extremely likely to participate in a future trial if they might be given a placebo	59.5	53.3	60.4	69.3	48.9	44.4
Respondents who would be very or extremely more willing to join a trial if an open label extension phase was offered	68.6	59.6	83.6	69.3	47.8	37.7
Respondents who felt it is either very or extremely important to have the results of a trial they participated in relayed back to them	84.5	87.1	87.5	83.3	81.3	78

### Level of evidence required before participating in a clinical trial

**Fig. 3:**
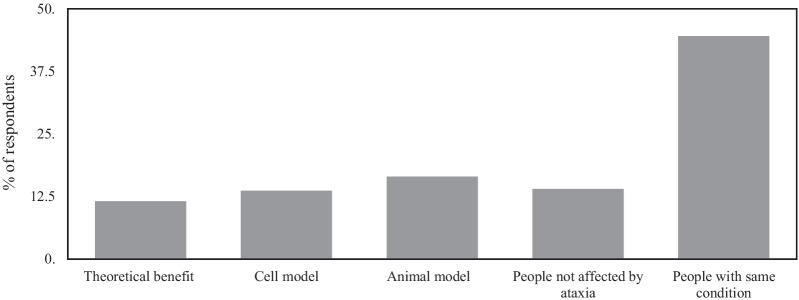
Level of evidence that respondents would be satisfied with before taking part in a clinical trial

We first asked respondents what the lowest acceptable level of evidence would lead to their trial participation. Across all cohorts, 44.6% of respondents wanted the drug to ‘have shown potential benefits in people with my condition’ with 16.5% wanting it to have shown benefit in an animal model of ataxia, 13.7% in a cell model of ataxia and 14% in people not affected by ataxia, with 11.6% of respondents content with only theoretical benefit (Fig. 3, Additional file 5: Table S4). We compared those who would accept a lower level of evidence (theoretical/cell model/animal model) to those who required a more rigorous level of evidence using a binary logistic regression model (see methods for variables used). Men were significantly more likely to accept a lower level of evidence (OR = 1.966, 95% CI 1.114, 3.470, *p* = 0.02) as were respondents who used walking aids (OR = 2.904, 95% CI 1.380, 6.114, *p* = 0.005) and respondents who had been in multiple trials (OR = 2.183, 95% CI 1.021, 4.667, *p* = 0.044).

### Drug repurposing

We asked respondents how likely they would be to enroll in a clinical trial if the medication had been proven safe for another unrelated condition. Of FRDA respondents, 75.2% would be ‘very’ or ‘extremely’ likely to enroll compared to 53.1% of inherited CA patients and 50.9% of idiopathic CA patients (Table 2).

### Intrathecal drug administration

To explore the acceptability of the intrathecal (IT) route of administration, we asked how often respondents would be prepared to have a lumbar puncture (LP) as a method of drug administration. Overall, 30.3% would never be prepared to accept this, 15.2% would be willing to have this done yearly, 19.5% 6 monthly, 10.5% every 2 months and 22.7% monthly (see Additional file 6: Table S5 for by-condition and subgroup breakdown).

In order to explore factors influencing willingness to undergo IT drug administration, we split the responses to the question into two groups: those who would be willing to have IT drug administration every 6 months or more frequently, and those who would never or only on a yearly basis. A binary logistic regression analysis was then conducted, using age groups, sex, condition, ambulatory status and whether or not they were interested in participating in a future trial as variables. Respondents who were interested in participating in a future trial were more likely to accept an LP every 6 months or more frequently (OR = 5.118, 95% CI 2.72, 9.450, *p* < 0.001). Other variables influencing willingness to undergo IT drug administration that displayed a trend towards significance were a diagnosis of FRDA (OR = 2.463, 95% CI 0.948, 6.401, *p* = 0.064) and using walking aids (OR = 2.140, 95% CI 0.962, 4.760, *p* = 0.062).

### Total time patients are prepared to dedicate to a clinical trial

Amongst respondents interested in taking part in a future trial (n = 178), we sought to determine the total amount of time which they would be willing to dedicate to it. To do this, we summed the amount of time respondents were willing to spend travelling to and from hospital, the amount of time they were willing to spend in hospital at each visit and multiplied this by the number of times they were willing to come to hospital in one year. This resulted in a mean (SD) value of 251.7 (181.6) hours. To investigate factors influencing this time dedication, we performed a multiple linear regression with dummy coding to allow for nominal variables. A model with the variables, age, sex, ambulatory status, condition and previous trial participation gave an R^2^ = 0.273 (F (12, 162) = 5.059, *p* < 0.001). Significant contributors to the model were having participated in more than 1 trial (B = 193.8, *p* < 0.001), age 26–35 (B = 96.49, *p* = 0.038), age 36–45 (B = 97.69, *p* = 0.035) and age 46–55 (B = 94.72, *p* = 0.037). Thus, those who had participated in more than one trial or were aged between 26 and 55 years would be willing to dedicate more time to a trial.

#### Procedures patients are willing to undergo during a trial

Most procedures were deemed acceptable by more than 70% of respondents with the exceptions of fasting (< 6 h = 68.6%, > 6 h = 53.2%), scans longer than one hour (64.5%), overnight stay in hospital (59.8%), muscle biopsy (49.2%) and lumbar puncture (42.7%) (Additional file 7: Table S6).

#### Reporting back trial results

Finally, we asked respondents to rate how important it was for them to have the results of any trial they participated in reported back to them and how they would prefer this to be done. Overall, 83.3% of respondents felt that it was either very or extremely important to have results reported back to them, with the most popular modes being through meeting with a physician (64.4%), and receiving a lay summary (54.2%) (Table 2).

## Discussion

Ours is the first study to provide comprehensive practical data from a large sample of ataxia patients, relating to the motivating factors for, and barriers to, clinical trial enrolment. Such data are essential to inform trial design and conduct by clinicians, researchers, advocacy organisations, regulators, and partners in the pharmaceutical industry.

Our cohort was multinational. As FARA focuses purely on FRDA, virtually all of the USA-based respondents had this diagnosis and, as such, we are unable to comment on the views of patients with inherited or idiopathic CA in the USA. Interestingly, both cohorts had a preponderance of female respondents (Table 1). To our knowledge there is no sex-bias in any of the ataxias represented here and given that there are no data demonstrating overrepresentation of women survey respondents [13] we find these data hard to explain.

Unsurprisingly, walking and balance problems were the most frequently selected symptoms across the entire cohort. This leads us to draw two main conclusions. Firstly, as neurorehabilitation programs have proven effective in the ataxias [14–16], directly impact the symptom respondents want addressed (mobility) and lack obvious detractors (side effects, invasive procedures and having to stop medication), more efforts might be expended in exploring this avenue of research. Secondly, current outcome measures such as the SARA and FARS include a subsection on gait, but this assessment is somewhat subjective. Given the importance of walking to patients, an outcome measure that accurately and objectively quantifies changes in gait and stance might be utilised in future trials. Several studies have validated the use of accelerometers in assessing physical activity levels [17, 18], in one case finding related data more responsive than SARA score in early stage spinocerebellar disease [19]. Such devices allow home study (reducing the cost and burden associated with travel to study appointments), might reduce inter-rater variability and, if used on a daily basis, might provide a much richer data set. Such wearable device use was deemed acceptable by 78.1% of the entire cohort.

Interestingly, the third most selected symptom across the whole cohort was fatigue, with a high ranking (6th on a list of symptoms with the biggest impact). This high figure is consistent with results from a 2016 Ataxia UK survey of 426 patients (JG personal communication). Fatigue impacts on quality of life and function in several ataxias [20–23] but, to our knowledge, only two clinical trials have used a specific fatigue rating scale as an outcome measure [24, 25]. We therefore suggest that future trials include a validated measure of fatigue as a secondary outcome measure, especially given the FDA’s preference for patient-reported outcome measures.

Assessment of symptoms that respondents would most like addressed by a trial showed a marked difference between wheelchair-dependent respondents, who selected dysarthria and dexterity more frequently than respondents who were still walking or walking with aids. Wheelchair-dependent respondents made up 54.6% of the FRDA cohort and, as such, represent a large pool of potential trial subjects if appropriate biomarkers are developed (i.e. that concentrate on speech, such as the Speech Intelligibility Test [26], and hand function [27]).

Factors motivating clinical trial participation were largely those relating to benefit to self and others. These motivations likely relate to patient/carer concerns about the lack of approved disease-modifying therapies and inexorable progression of ataxic conditions. This desire for a cure may also explain the striking percentage of patients willing to accept what the scientific community would consider low-level evidence for a trial medication.

Physician input was also of clear importance for many respondents, a finding recapitulated in other conditions [28, 29]. Interestingly, this seemed more important to those who were wheelchair-dependent, which may reflect increased healthcare needs in more advanced stages of disease or that they may have had a longer relationship with the physician in question.

Travel burden, both in terms of distance and cost, was a clear factor influencing trial participation in this group of subjects where mobility is often severely affected. Once again, cost reimbursement or availability of a travel assistance coordinator will likely improve recruitment. Additionally, consideration of use of wearable devices for home data collection, as discussed above, will reduce travel burden for trial participants.

Other deterrents to trial participation common across all three conditions were fear of side effects and concerns about stopping current medications. Data are lacking about the frequency of adverse events directly related to the study drug in clinical trials, partly due to variation in the reporting measures [30, 31]. It is thus hard to give potential participants a true estimate of how common such events might be. Stopping current medication may be a requirement for participation in certain trials, and our data suggest that this deterrent is more important for those patients at a more advanced disease stage. It is therefore incumbent on those organising a trial to make it explicit that participants may have to stop certain medications, but that the use of often-available alternative medications can be discussed with the trial team.

Amongst our entire cohort, only 57.1% would be willing to enroll in a clinical trial if there were a chance that they would be given a placebo, although this percentage was higher if an open label extension phase was offered. This sentiment varied with previous trial participation and whether the respondent used walking aids. This variance might be explained by self-selection bias, or by the education in trial design that patients often receive whilst taking part in trials. This issue with placebos is found in many other conditions [32, 33] and could lead to selection bias. However, when properly addressed with appropriate information [34], the majority of patients deemed placebo randomisation to be acceptable. There is thus an onus on the ataxia research community to enhance education about clinical trials, if recruitment [35] and retention are to be improved. Additionally, alterations to trial design could be used to work around this problem; unequal drug:placebo randomisation has been commonly used [36] (although this has its detractors [37]), as have novel trial designs such as multi-arm, parallel group studies [38] with other designs even more applicable to rarer diseases [39]. Open label extensions have also been used to increase participation in trials, and although there are questions about their benefits [40], our FRDA subjects were attracted by such design.

A remarkable percentage of respondents would participate in a trial on the basis of what the scientific community would consider relatively sparse pre-clinical data. There is already a large body of literature indicating that, in many circumstances, men are more likely to take part in risky behaviour than women [41] and our data concur with this, with men more likely to participate in trials with lower levels of pre-clinical data. We also demonstrate that patients who are dependent on walking aids are more likely than other groups to participate in trials with limited pre-clinical evidence. We hypothesise that this may be due to the concerns surrounding potentially imminent wheelchair dependence, something that in our clinical experience patients strive to avoid for as long as possible. These data have implications for organisations with oversight of study development and trial conduct such as national trial regulators, institutional review boards and independent ethics committees when they adjudge how much data is required before taking drugs into human trials. We stress, however, that our results must be also considered in the context of respondents’ limited understanding of the pre-clinical phase of drug development.

Regarding the phase of trial that respondents would be most likely to participate in, we found phase 2 trials to be the most popular in FRDA and idiopathic CA. Possible explanations for this include the limited time commitment (which was highlighted in our explanation of different trial phases), frequent absence of placebo use and confidence in established safety data from a Phase 1 study.

Overall, 53.8% of respondents considered IT drug every 6 months or more frequently to be acceptable, and 69.7% on an annual basis. Those interested in future trials were significantly more likely to accept such intervention. These results are important for trials of anti-sense oligonucleotides (ASOs), which currently require IT injection. Frequency of delivery is also a key factor. Phase 1 ASO trials have tended to use once-only dosing [42, 43], while later phase studies have used varied dosing regimens [36, 44]. Any future trial of IT drug delivery would do well to educate prospective participants about the safety and tolerability [45, 46] of lumbar puncture, as this procedure still clearly concerns patients.

This study has some limitations. Given that participation in the survey was voluntary and unremunerated, it seems unlikely that patients would provide spurious data, although no formal validation of data accuracy was undertaken. As the overall proportion of respondents with FRDA from the U.K. and the U.S. is 59.6%, their views will tend to override those of respondents affected by CA. We did not ask specifically which type of inherited CA respondents had, as we were concerned that the numbers of each type of CA diagnosis would be so few as to make analysis meaningless. It is our hope that through studies such as European Spinocerebellar ataxia type-3 Machado Joseph Disease Initiative (ESMI) and European integrated project on spinocerebellar ataxias (EUROSCA), their views on trials can be further explored.

## Conclusion

We consider these data to be of great importance for those designing trials in the field of ataxia, and to the regulatory agencies and ethics committees upon whom rests the eventual decision as to whether a trial can proceed. We also hope that this study might encourage others working in rare neurodegenerative diseases to gather the opinions of their patients in order that they too may benefit from their perspective on trial designs.

## Patients and methods

We designed and conducted an online electronic survey to determine patients attitudes towards clinical trials in adult ataxia patients.

### Participants

Participants were aged > 16 years with a self-reported diagnosis of ataxia, this study therefore included minors according to US and UK law. However in the UK young people aged 16–18 years with sufficient understanding are able to give their full consent to participate in research independently of their parents and guardians. They were recruited via two patient support organisations: Ataxia UK (based in the UK) and the Friedreich's Ataxia Research Alliance (FARA, based in the United States of America (USA)). Ethical approval was granted by the Research Ethics Committee and Health Research Authority in the United Kingdom (IRAS ID: 260860, REC Ref: 19/LO/0764) and the Institutional Review Board at the Children’s Hospital of Philadelphia in the USA (IRB ID: 19-016104). When patients were unable to complete the questionnaire themselves, parents, partners or caregivers did so on their behalf (22.8% of all responses) and provided their consent.

### Survey design

The survey was informed by informal interviews conducted by two authors (GTB and AD) at the National Hospital for Neurology and Neurosurgery, London, UK with a convenience sample of six Friedreich's ataxia patients, three ataxia specialist clinicians and three clinical trial staff over the course of two weeks. The resultant draft survey was reviewed by Ataxia UK and FARA and amended in response to their comments, ultimately yielding 29 non-obligatory questions relating to four key domains (demographics, personal motivation, drug therapy and study design), and a feedback box. Response options varied by the type of questions and ranged from numerical, categorical and ordinal (Likert scale) to open ended comments. All medical terms in the survey were highlighted and lay explanations were placed below the question (See survey form in Additional file 7: Appendix 1).

### Data collection

Ataxia UK and FARA emailed a brief introduction to the study and the survey link to eligible individuals (patient; or their carer, parent or partner) in their mailing lists (Ataxia UK ~ 3000 individuals, FARA/Children Hospital of Philadelphia (CHOP) ~ 500 individuals), and posted it on their social media pages. Ataxia UK also published an advertisement in The Ataxia magazine. The Children's Hospital of Philadelphia, a FARA collaborator, also sent the survey-link to their FRDA patient contact list. Reminder emails were sent periodically to increase participation. Before completing the survey, participants were invited to read the patient information sheet (PIS), and then provided informed consent. The consent form was designed as a question whereby participants who answered 'yes' could proceed to the main survey and participants who replied 'no' would have the session terminated. Two different PIS and consent forms were created due to differences in requirements of regulatory approval bodies between the USA and UK. All the questions wer e identical between the two surveys.

### Statistical analyses

Any questions to which a respondent gave contradictory responses were removed from the analysis. Incomplete surveys were not removed from the analysis, as respondents chose to answer some questions and to skip others. The number of respondents was reported for each individual question. Responses to those questions in which a respondent selected more than the maximum number of options were excluded from the analysis. Since the number of respondents differed for each group depending on the question, we presented the results in percentage when appropriate.

In questions with responses measured along a 5-point Likert scale, affirmative responses were considered to be either `very interested/likely/willing’ or `extremely interested/likely/willing' for all categories. Results were prepared as tabulated descriptive statistics and presented as numbers (n) and percentage (%) of total respondents per question.

Responses were grouped according to condition (FRDA, inherited cerebellar ataxia, idiopathic cerebellar ataxia). Subgroup analysis was performed for age ranges (< 15–25, 26–35, 36–45, 46–55, 56–65 and > 65 years); three different ambulatory statuses (walking independently, walking with aids (use of a stick/ two sticks/ wheelchair when outdoors) and wheelchair dependent); and, for FRDA only, previous trial experience (trial naive, experience of one trial, experience of multiple trials). We did not include the idiopathic and inherited cerebellar ataxia groups in the analysis on previous trial experience as the number of participants who had been on a trial for each group was insufficient (n = 4 and n = 5 respectively).

To explore the reasons behind the choices made, responses of interest were split into two groups (see text below) and binary logistic regression analysis used to investigate the influence of the variables age, sex, condition, ambulatory status and previous trial exposure on interest in future trial participation. Linear regression analysis was also used to investigate influences on total time commitment to trial.

## Supplementary Information

**Additional file 1:** Survey questionnaire.

**Additional file 2:** Symptoms patients want addressed.

**Additional file 3:** Reasons for trial participation.

**Additional file 4:** Barriers to trial participation.

**Additional file 5:** Level of evidence.

**Additional file 6:** Lumbar puncture frequency.

**Additional file 7:** Trial design.

## Data Availability

The datasets generated during and/or analysed during the current study are available from the corresponding author on reasonable request.
