# Supplementary material for: The attitude of patients with progressive ataxias towards clinical trials
Source: Orphanet J Rare Dis. 2022 Jan 4;17:1. doi: 10.1186/s13023-021-02091-x (PMC8729009; doi:10.1186/s13023-021-02091-x)
Supplement: Supplementary file 1 — Additional file 1: Survey questionnaire. [file 13023_2021_2091_MOESM1_ESM.pdf]

The attitudes of patients with ataxia to clinical trials - UK survey

1. Consent form

IRAS ID: 260860

REC Ref: 19/LO/0764

* 1. I confirm that I have read the information sheet dated 22/03/2019 (version 1) for the above study. I have had the opportunity to consider the information, ask questions and have had these answered satisfactorily.

I understand that my participation is voluntary and that I am free to withdraw at any time without giving any reason, without my medical care or legal rights being affected.

I understand that the information collected about me will be used to support other research in the future, and may be shared anonymously with other researchers.

I agree to take part in the above study.

By ticking "Yes" you agree to the above conditions and will proceed to the survey.
Ticking "No" will terminate the session.

0

- ◯ Yes
- ◯ No

If you have any questions about the research study, please contact either:

Julie Greenfield.
Email: jgreenfield@ataxia.org.uk
Telephone: 020 7582 1444
Address: Ataxia UK. 12 Broadbent Close, London, N6 5JW.

or:

Dr Gilbert Thomas-Black
Telephone: 020 3448 3100
Email: g.thomas-black@ucl.ac.uk
Address: The National Hospital for Neurology and Neurosurgery
Department of Clinical and Movement Neurosciences
Queen Square, London
WC1N 3BG 0

2. **Demographics**

This is the first section of the questionnaire. There are 4 sections in total.

Medical terms in blue and marked with (*) are explained below the questions.

2. Are you a …? (choose one) 0

- Patient
- Carer
- Parent
- Partner

If you have answered anything other than patient, please complete the remainder of the questionnaire from the point of view of the patient. 0

3. What is your age? (choose one) 0

- Under 15
- 15 – 25
- 26 – 35
- 36 – 45
- 46 – 55
- 56 – 65
- Over 65

4. What best describes your gender? (choose one) 0

- Male
- Female
- Other (please specify)

5. Which ataxia do you have? (choose one) 0

- Friedreich's ataxia
- Inherited cerebellar ataxia
- Cerebellar ataxia (unknown cause)
- Episodic ataxia
- Other (please specify)

6. Which of the following best describes how your ataxia affects your mobility? (choose one) 0

- ◯ No functional impairment
- ◯ Mild, able to run, walking unlimited
- ◯ Moderate, unable to run, limited walking without aid
- ◯ Walking with one stick
- ◯ Walking with two sticks
- ◯ Walking with aid of walker indoors, utilising wheelchair outdoors
- ◯ Unable to walk, requiring wheelchair
- ◯ Confined to bed

7. Have you ever signed up for or participated in any way in a medication clinical trial? (choose one) 0

- ◯ Yes - once
- ◯ Yes - twice
- ◯ Yes - 3 times
- ◯ Yes - more than 3 times
- ◯ No

8. Have you completed a medication clinical trial? (choose Y/N; if No choose all applicable) 0

- ☐ Yes
- ☐ No - Side effects prevented me
- ☐ No - Was told to withdraw by clinical trial staff
- ☐ No - Financial burden too high
- ☐ No - Procedures were too invasive
- ☐ No - Did not feel valued
- ☐ N/A
- ☐ Other (please specify)

The attitudes of patients with ataxia to clinical trials - UK survey

3. Trial design preference

This is the second section of the questionnaire.

Medical terms in blue and marked with (*) are explained below the questions.

9. If you have participated in a medication clinical trial, what could be improved about the *Patient Information Sheet (PIS)? (choose all that apply) 0

- [ ] Language – too technical
- [ ] Length – too long
- [ ] Have the possibility to discuss the PIS with a physician/ healthcare professional involved in the clinical trial
- [ ] Other (please specify)
- [ ] Nothing – PIS was satisfactory
- [ ] N/A

**The PIS is a leaflet which the researchers are obligated to provide to participants to make sure the participants are giving informed consent. The leaflet should contain information about why the research is being done, how long it will last, and what methods will be used, the possible risks and benefits, what taking part will practically involve, who to contact and data privacy.* 0

10. Would you be interested in participating in a medication clinical trial in the future? (choose one) 0

- Not at all interested
- A little interested
- Somewhat interested
- Very interested
- Extremely interested

11. How likely would you be to participate in a medication clinical trial if you might be given a *placebo? (choose one) 0

- Not at all likely
- A little likely
- Somewhat likely
- Very likely
- Extremely likely

**A placebo is an inactive substance, typically a tablet or a capsule which does not contain an active drug ingredient. For example, placebo pills or liquids may contain starch, sugar, or saline. Placebos are often used in clinical trials as an inactive control so that researchers can evaluate the true overall effect of the medication being studied.* 0

12. What level of evidence would satisfy you before joining a medication clinical trial? (choose one) 0

- Theoretical benefit - no experimental proof
- Some benefit demonstrated in a cell model of ataxia
- Some benefit demonstrated in an animal model of ataxia
- Shown to be safe in people not affected by ataxia
- Shown to have potential benefits in people with my condition

13. Which phase clinical trial would you participate in? (choose all that apply) 0

- [ ] Phase I – 1-10 participants, several months, focused on safety and dosage, as well as method of administration
- [ ] Phase II – 20-30 participants, several months, focused dosage and effectiveness in preventing or treating condition
- [ ] Phase III – hundreds of participants, can last up to several years usually *double-blind design, focus on proving efficacy compared to current treatment or **placebo and collecting long term safety data

**A double-blind trial is one in which neither the participants nor the experimenters know who is receiving the treatment or the placebo. This procedure is used to prevent bias in research, and it is seen as the "gold standard" of medical trials.*

***A placebo is an inactive substance, typically a tablet or a capsule which does not contain an active drug ingredient. For example, placebo pills or liquids may contain starch, sugar, or saline. Placebos are often used in clinical trials as an inactive control so that researchers can evaluate the true overall effect of the medication being studied.* 0

14. If a study medication had already been proven safe for an unrelated condition, how likely would this make you to enrol in a clinical trial? (choose one) 0

- Not at all likely
- A little likely
- Somewhat likely
- Very likely
- Extremely likely

15. Would the opportunity to participate in an *open label extension to the trial make you more willing to participate in a **placebo – controlled medication clinical trial? (choose one) 0

- Not at all willing
- A little willing
- Somewhat willing
- Very willing
- Extremely willing

**An open-label extension trial, is a type of clinical trial in which both the researchers and participants know which treatment is being administered as all the participants are given the study drug. The objective is to gather information about safety and tolerability of the new drug in long term, day to day use. This type of trial commonly succeeds Phase 3 trials.*

***A placebo is an inactive substance, typically a tablet or a capsule which does not contain an active drug ingredient. For example, placebo pills or liquids may contain starch, sugar, or saline. Placebos are often used in clinical trials as an inactive control so that researchers can evaluate the true overall effect of the medication being studied.* 0

16. Which symptoms would you like most to be addressed by a medication clinical trial? (choose 3) 0

- [ ] Balance problem/unsteadiness
- [ ] Walking
- [ ] Clumsiness
- [ ] Falling
- [ ] Slurred speech/dysarthria
- [ ] Standing
- [ ] Using hands/dexterity
- [ ] Tiredness/fatigue/lack of stamina
- [ ] Bladder incontinence
- [ ] Coughing/choking
- [ ] Weakness
- [ ] Dizziness
- [ ] Swallowing
- [ ] Cardiomyopathy/ heart problems
- [ ] Vision
- [ ] Hearing

4. **Trial burden preference**

This is the third section of the questionnaire.

Medical terms in blue and marked with (*) are explained below the questions.

17. Would you be willing to enter into a medication clinical trial if the medication was regularly administered… (choose all that apply) 0

- [ ] In the form of a pill/ liquid/ powder?
- [ ] Infused into the blood stream via hollow plastic needle inserted into a vein?
- [ ] Injected into the fluid around the spinal cord via a needle going into the lower back?

18. How often would you be prepared to get a *lumbar puncture as method of drug administration in the trial? 0

- ○ Once / month
- ○ Once / 2 months
- ○ Once / 6 months
- ○ Once / 12 months
- ○ Never

**A lumbar puncture is a procedure in which a hollow needle is inserted between the bones of the lower back and into the fluid around the lower part of the spinal cord. This procedure can be used to collect a sample of cerebrospinal fluid for examination. The procedure itself should not be painful but there might be headaches and back pain the following days.* 0

19. What is the maximum length of **each visit** you would be willing to come in to hospital for in a medication clinical trial? (choose one) 0

- ○ 2 hours
- ○ 4 hours
- ○ 6 hours
- ○ 8 hours
- ○ 2 days

20. Considering your answer to question 19, in a **6-month** long medication trial please select the maximum visit frequency to hospital that would be an acceptable time commitment for you. (choose one) 0

- ○ Twice
- ○ 4 times
- ○ 8 times
- ○ 12 times

21. Considering your answer to question 19, in a **12-month** long medication trial please select the maximum visit frequency to hospital that would be an acceptable time commitment for you. (choose one) 0

- Twice
- 4 times
- 8 times
- 12 times

22. Considering your answer to question 19, in a **24-month** long medication trial please select the maximum visit frequency to hospital that would be an acceptable time commitment for you. (choose one) 0

- Twice
- 4 times
- 8 times
- 12 times
- 24 times

23. What is the maximum one-way length of travel door to door you would be prepared to undergo to participate in a medication clinical trial? (choose one) 0

- 30 minutes
- 1 - 2 hours
- 3 - 4 hours
- 5 – 6 hours
- 7 – 8 hours
- 1 day

24. Which of the following procedures would you feel comfortable undergoing as part of a medication clinical trial? (select all which are applicable) 0

- [ ] Telephone conversation with doctor
- [ ] Respond to questionnaire
- [ ] At - home monitoring of vital signs eg. using Fitbit or Apple watch
- [ ] Neurological examination in hospital
- [ ] Urine sample in hospital
- [ ] Blood test in hospital
- [ ] Fasting <6 hours
- [ ] Fasting >6 hours
- [ ] *Scans (eg. MRI, CT, PET) <1 hour
- [ ] *Scans (eg. MRI, CT, PET) >1 hour
- [ ] Overnight stay in hospital
- [ ] **Muscle biopsy
- [ ] ***Lumbar puncture
- [ ] Exercise test (eg. seated bike)

** Scans refer to medical imaging techniques which create visual representations of the interior of a body for medical interpretation. These scans normally involve the patient lying still for prolonged periods of time which is not painful but can become uncomfortable. Sometimes there might be a need for an injection with a contrasting agent.*

***A muscle biopsy is a minor surgical procedure, which involves removing a small piece of muscle for analysis. There is usually little or no pain during this procedure, but an uncomfortable tugging may be felt.*

****A lumbar puncture is a procedure in which a hollow needle is inserted between the bones of the lower back and into the fluid around the lower part of the spinal cord. This procedure can be used to collect a sample of cerebrospinal fluid for examination. The procedure itself should not be painful but there might be headaches and back pain the following days.*

0

25. What is the maximum severity side-effects you would be prepared to experience during a medication clinical trial? (choose one) 0

- ○ No side effects
- ○ Mild - e.g. headache or a mild upset stomach
- ○ Moderate - e.g. diarrhea and vomiting or an itchy rash
- ○ Severe - e.g. requiring hospitalization
- ○ Life-threatening - e.g. requiring intensive care

5. **Trial result feedback**

This is the final section of the questionnaire.

26. What are the top factors that would make you more likely to participate in a medication clinical trial? (select maximum 6) 0

- [ ] Potential benefit to self
- [ ] Potential benefit to others
- [ ] Feeling of increased care/obtaining extra health check ups
- [ ] Feeling valued
- [ ] Financial reimbursement of expenses (covering costs of travel and accommodation if applicable)
- [ ] Recommendation of trial by physician
- [ ] Recommendation of trial by patient support organization
- [ ] Recommendation from friend with same condition
- [ ] Social media advertising
- [ ] Person of contact within clinical trial team
- [ ] Availability of physician should there be any issues
- [ ] Webinar to discuss trial before starting
- [ ] Other (please specify)

27. What factors would make you less likely to join a medication clinical trial? (select all that are applicable) 0

- [ ] Stopping current regular medication
- [ ] Not being able to take future medication which might interact with drug tested during trial
- [ ] Burden of travel
- [ ] Costs associated with travel
- [ ] Unsupportive peers and families
- [ ] Clinical trial overlaps with important life event
- [ ] Having to miss work/school
- [ ] Fear of consequences of dropping out
- [ ] Having to change diet / lifestyle
- [ ] Worried clinical trial drug is not effective
- [ ] Fear of side effects

28. How important is it to learn the results of the medication clinical trial you have participated in? (choose one) 🗨 0

- ○ Not at all important
- ○ A little important
- ○ Somewhat important
- ○ Very important
- ○ Extremely important

29. If you wanted to learn the trial results how often would you like them to be conveyed back to you? (choose one) 🗨 0

- ○ Interim updates
- ○ Final summary

30. How would you like the results to be conveyed back to you? (choose all that apply) 🗨 0

- ☐ Lay summary
- ☐ Entire medical journal paper
- ☐ Webinar
- ☐ Meeting with physician
- ☐ Conference presentation

31. Please leave any additional comments if you wish 🗨 0

Thank you for completing our survey!
If you have answered all the questions you wish to, please click "Submit". 🗨 0
